# Ankle perturbation generates bilateral alteration of knee muscle onset times after unilateral anterior cruciate ligament reconstruction

**DOI:** 10.7717/peerj.5310

**Published:** 2018-07-31

**Authors:** Patricio A. Pincheira, Rony Silvestre, Susan Armijo-Olivo, Rodrigo Guzman-Venegas

**Affiliations:** 1 Escuela de Kinesiología, Facultad de Ciencias, Universidad Mayor, Santiago, Chile; 2 Centre for Sensorimotor Performance, School of Human Movement and Nutrition Sciences, University of Queensland, Brisbane, Australia; 3 Unidad de Biomecánica Deportiva, Clinica Meds, Santiago, Chile; 4 Department of Physical Therapy, Faculty of Rehabilitation Medicine, University of Alberta, Edmonton, Canada; 5 Laboratorio Integrativo de Biomecánica y Fisiología del Esfuerzo (LIBFE), Escuela de Kinesiología, Facultad de Medicina, Universidad de los Andes, Santiago, Chile

**Keywords:** Surface electromyography, Anterior cruciate ligament reconstruction, Proprioception, Neuromuscular control

## Abstract

**Background:**

The aim of this study was to compare muscle activation onset times of knee muscles between the involved and uninvolved knee of patients with unilateral anterior cruciate ligament reconstruction (ACLR), and the uninjured knees of healthy subjects after a controlled perturbation at the ankle level.

**Methods:**

Fifty male amateur soccer players, 25 with unilateral ACLR using semitendinosus-gracilis graft (age = 28.36 ± 7.87 years; time after surgery = 9 ± 3 months) and 25 uninjured control subjects (age = 24.16 ± 2.67 years) participated in the study. Two destabilizing platforms (one for each limb) generated a controlled perturbation at the ankle of each participant (30°of inversion, 10°plantarflexion simultaneously) in a weight bearing condition. The muscle activation onset times of semitendinosus (ST) and vastus medialis (VM) was detected through an electromyographic (EMG) analysis to assess the neuromuscular function of knee muscles.

**Results:**

Subjects with ACLR had significant delays in EMG onset in the involved (VM = 99.9 ± 30 ms; ST = 101.7 ± 28 ms) and uninvolved knee (VM = 100.4 ± 26 ms; ST = 104.7 ± 28 ms) when compared with the healthy subjects (VM = 69.1 ± 9 ms; ST = 74.6 ± 9 ms). However, no difference was found between involved and uninvolved knee of the ACLR group.

**Discussion:**

The results show a bilateral alteration of knee muscles in EMG onset after a unilateral ACLR, responses that can be elicited with an ankle perturbation. This suggests an alteration in the central processing of proprioceptive information and/or central nervous system re-organization that may affect neuromuscular control of knee muscles in the involved and uninvolved lower limbs.

## Introduction

Anterior cruciate ligament (ACL) injuries are common in sporting activities, and are of concern in orthopedic and sports medicine (Kaeding, Léger-St-Jean & Magnussen, 2017). Close to 70–80% of all ACL injuries occur without direct contact from another person (Agel, Arendt & Bershadsky, 2005; Kobayashi et al., 2010; Kaeding, Léger-St-Jean & Magnussen, 2017), where a ligament is strained or torn as a result of an unexpected perturbation. ACL injuries subsequently lead to a myriad of effects, including altered knee kinematics during movement (Hébert-Losier et al., 2018), strength loss (Gumucio et al., 2018), and reduced sensorimotor information (Nyland et al., 2017). The primary reason for these complex secondary effects is the loss of knee joint stability (Kvist, 2004), subsequent to an impairment in the neuromuscular control of the lower limb muscles (Bryant, Kelly & Hohmann, 2008; Dingenen et al., 2015). Furthermore, this neuromuscular deficit may persist even following an ACL reconstruction (ACLR) (Dingenen et al., 2016).

As a result of tissue damage associated with ACL surgery and graft preparation (Tadokoro et al., 2004; Takeda et al., 2006), changes in sensory information and neuromuscular control could occur (Riemann & Lephart, 2002), resulting in deficits in dynamic control and joint stability (Madhavan & Shields, 2011). Moreover, central nervous system (CNS) neuroplastic changes (Grooms, Appelbaum & Onate, 2015; Grooms et al., 2017), reorganization in sensorimotor cortical areas (Grooms et al., 2017; Nyland et al., 2017; Needle, Lepley & Grooms, 2017) and/or synaptic coupling between mechanosensitive ligament afferents with the *γ*-muscle spindle system (Sjölander, Johansson & Djupsjöbacka, 2002; Konishi, Konishi & Fukubayashi, 2003) may be involved in the neuromuscular impairment of knee muscles bilaterally. Therefore, investigating muscle activation patterns in the contralateral non-injured leg of ACLR patients may help to reveal the role of CNS adaptations after ACL injury and reconstruction (Dingenen et al., 2015; Dingenen et al., 2016; Grooms et al., 2017; Needle, Lepley & Grooms, 2017).

It has been shown that after an ACLR, quadriceps and hamstring muscles present deficits in neuromuscular control (Bryant, Kelly & Hohmann, 2008; Bryant, Newton & Steele, 2009; Madhavan & Shields, 2011). Altered activation patterns in these muscles have been found not only in the involved knee (Madhavan & Shields, 2011), but also in the contralateral uninjured knee (Lustosa et al., 2011; Dingenen et al., 2016). Further, a delayed reaction of quadriceps and hamstrings in response to a perturbation may contribute to impaired postural control and joint stability (Nyland et al., 2017). Vastus medialis (VM) weakness (Marcon et al., 2015) and altered neuromuscular excitability (Rosenthal et al., 2009), are frequently found in ACLR patients even a year after completion of their rehabilitation program (Marcon et al., 2015). Semitendinosus (ST) muscle when used for graft purposes, presents clear changes in EMG activation (Makihara et al., 2006) and electromechanical delay (Ristanis et al., 2009) after the surgery. These impairments are often exaggerated during a perturbation (Madhavan & Shields, 2011), offering a useful window to explore the physiological basis of neuromuscular control of the lower limb (Malfait et al., 2015).

Regarding lower limb neuromuscular control, ankle proprioception appears to be the most critical for balance control contributing to sport performance (Han et al., 2015). Because during most sports activities, the ankle-foot complex is the only part of the body in contact with the ground, ankle proprioception provides essential information to enable adjustment of ankle positions and movements of the body in order to successfully perform the motor tasks required (Han et al., 2015). Further, stretch and force-sensitive receptors from the ankle joint may modulate knee muscles activation/inhibition (Gueguen et al., 2005), contributing to lower limb inter-segmental synergies under central command (João et al., 2014). Given that ankle sensory information relates with brain activity during balance performance (Goble et al., 2011), this information should be considered in the evaluation of patients with ACL injury and reconstruction, where CNS neuroplastic changes and reorganization play a crucial role (Grooms, Appelbaum & Onate, 2015; Dingenen et al., 2015; Dingenen et al., 2016; Needle, Lepley & Grooms, 2017).

The aim of this study was to investigate the muscle activation onset latencies of the vastus medialis (VM) and semitendinosus (ST) muscles during an inversion perturbation that applies stress in the ankle joint. We wanted to see the neuromuscular behavior of these muscles after subjects returned to sport activities following an ACLR. Specifically, our goals were: (i) to compare the onset times of the VM and ST muscles between the knee that underwent ACLR (i.e., involved knee) with the uninvolved leg of healthy subjects; (ii) to compare the onset times between the uninvolved knee of subjects who underwent ACLR and uninvolved healthy subjects; (iii) to compare the onset times of the involved knee with the contralateral uninvolved knee in the ACLR group. It has been suggested that changes in muscle activation patterns, in particular the altered timing of muscle onset, might be undesirable in ACLR patients returning to normal activities (Crow, Pizzari & Buttifant, 2011). The onset of EMG activity is a measure that can assist in the evaluation of neuromuscular function (Hodges & Bui, 1996). Although this parameter has been used in studies to characterize subjects with ACL deficiency (Beard et al., 1994; Madhavan & Shields, 2011; Dingenen et al., 2015), few studies have examined bilateral activation changes in the knee muscles after a unilateral ACLR (Dingenen et al., 2016). Distal perturbations in a weight bearing situation provides an appropriate measurement of dynamic neuromuscular control of the lower limb (Malfait et al., 2015), approximating the test to real life settings, thus increasing the ecological validity of the evaluation (Han et al., 2015; Han et al., 2016). This simple paradigm may be useful to provide new insights into neuromuscular control strategies that contribute to joint stability after ACLR (Malfait et al., 2015). Given that previous studies suggests bilateral neuromuscular deficiencies during walking (Lustosa et al., 2011) and during transition tasks (Dingenen et al., 2016), we hypothesized that ankle perturbations will reveal bilateral alterations in the onset of EMG activity of the VM and ST muscles.

**Table 1:** Characteristics of control and ACLR groups (*N* = 50).

	Group	Mean	Std. Deviation	*N*
Weight (Kg)	ACLR	77.56	6.35	25
	Healthy	78.16	5.46	25
Height (cm)	ACLR	169	7	25
	Healthy	172	5	25
BMI	ACLR	26.99	1.39	25
	Healthy	26.34	1.23	25
Age (years)	ACLR	28.36	7.87	25
	Healthy	24.16	2.67	25
Time from surgery (months)	ACLR	9	3	25
	Healthy	–	–	–

**Notes.**

ACLR anterior cruciate ligament reconstruction

There were no significant differences between the groups in any of the variables listed (*p* > 0.05).

## Methods

### Participants

This study incorporated a multiple cross-sectional design including two groups of participants: subjects with unilateral ACLR and healthy controls. Based on a difference in onset latencies between groups of 25 ms (Beard et al., 1994), which is equivalent to a large effect size (*d* = 0.9), a sample size of 23 subjects per group was required (*α* = 0.05 and *β* = 0.20). Fifty subjects (25 healthy, 25 with ACLR) were recruited using the following inclusion criteria (both groups): amateur soccer players (playing at least twice a week), male, between 18 and 40 years, without diagnosed neuromuscular problems (e.g., neuropathy, stroke). Subjects were excluded from the study if they had: (1) a previous surgical intervention in either of the lower extremities; (2) a history of ankle musculoskeletal impairments (e.g., ankle sprain) in the last 6 months; (3) a history of ankle instability (more than two ankle sprains in the same ankle in the same year, and/or subjects reporting subjective feeling of ankle instability); (4) acute or chronic pain in the lower extremities in the past 6 months; (5) had less than 6 months of evolution from surgery; (6) more than one concomitant ligament injury/repair at the time of surgery; or (7) had a body mass index higher than 30 (kg/m^2^). Demographics and sample characteristics of both groups are detailed in Table 1. Subjects with ACLR were on average 9 ± 4 months post-surgery at the time of this study. The control group was selected from students and staff at the university. Subjects with ACLR were recruited from the trauma service of the university hospital. The injury in all subjects was caused by a non-contact mechanism during a soccer match on the dominant limb. The time between ACL injury and surgery was on average 3.4 ± 1 months for the study group. All subjects had undergone a unilateral arthroscopic reconstruction with the same surgical team at least six months before the study, using ipsilateral semitendinosus-gracilis grafts. Five subjects in the study group had one concomitant injury repaired in the same ACLR surgery (e.g., posterior cruciate ligament). Detailed information of concomitant repairs for each individual is presented in the supplementary material. Post-surgery, the subjects followed and completed a conventional rehabilitation program in both limbs as recommended in the literature (Kvist, 2004). All ACLR subjects were cleared to participate in sports activities by an orthopedic surgeon with more than 15 years of experience, based on their perceived functional status, patient reported outcomes, and functional-clinical evaluation (e.g., strength, range of motion, anterior drawer test). At the moment of evaluation, subjects were able to perform plyometric exercises, jumping, and cutting activities. However, patients were advised to get back to amateur full time athletic competition (high contact) gradually. The study was approved by the local university ethics committee (Universidad Mayor, CRI 108, 772018) and conducted according to the Declaration of Helsinki.

**Figure 1:**
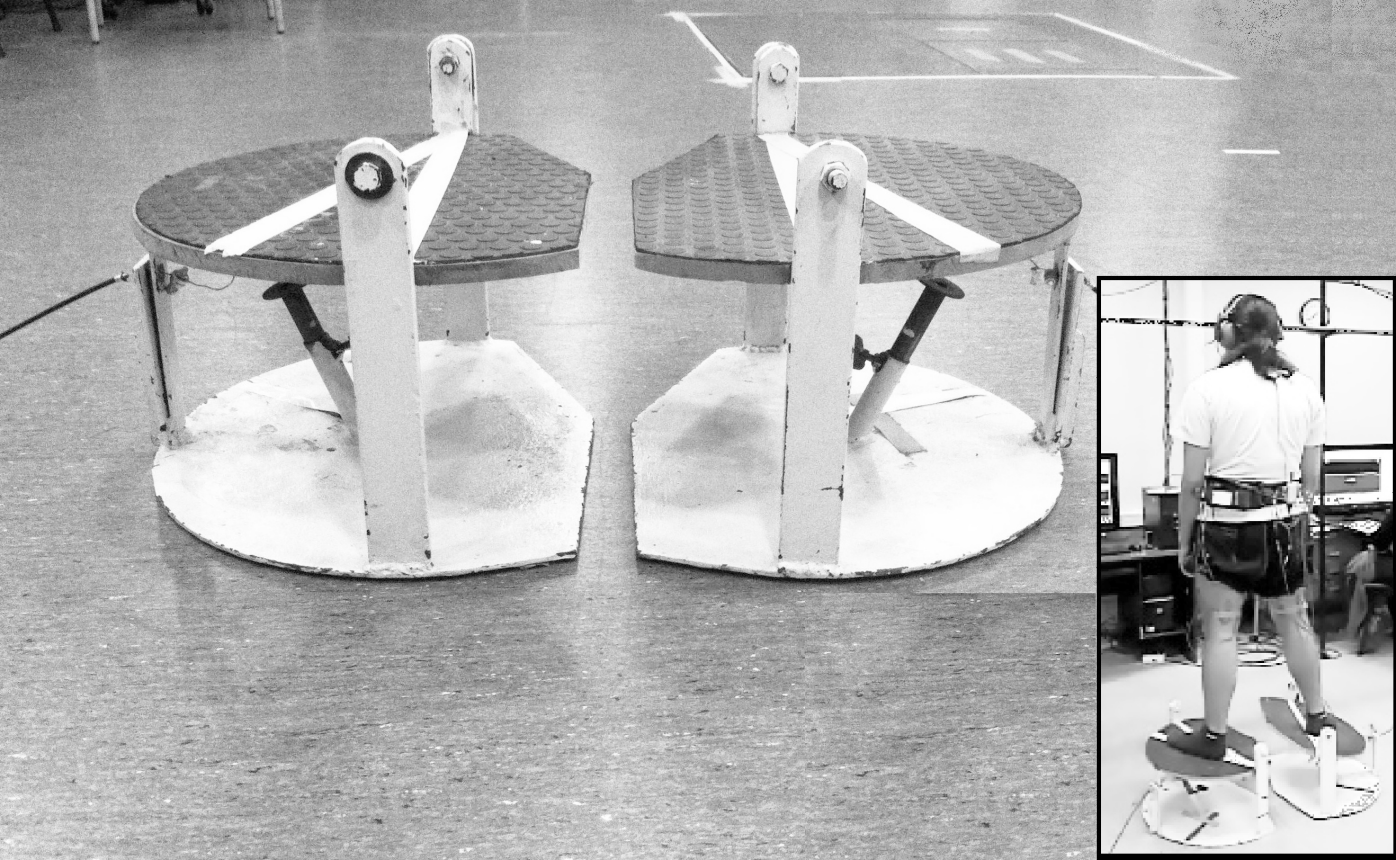
Platform used to elicit the perturbation at the ankle level while standing. When the device is activated by the operator, a sudden fall driven by the force of gravity generates 30°of inversion in the frontal plane and to 10°of plantar flexion in the sagittal plane. In the inset, a subject with the mechanism released at the right limb. Photo credit: Patricio A. Pincheira.

### Procedures: perturbating platforms

Two destabilizing platforms (one for each limb) integrated in an ankle perturbation device (Riemann, Myers & Lephart, 2002) were used to generate a perturbation of each individual’s base of support. These platforms, through an unexpected and random sudden fall driven by the force of gravity, generate a perturbation to the subjects at the ankle level in two planes simultaneously by moving the base of support to 30°in the frontal plane (inversion) and to 10°in the sagittal plane (plantarflexion) (Fig. 1). The time between the release and the stop (impact) of the mechanism was 200 ± 10 ms. This applies stress on the lateral ligamentous complex of the ankle also generating an external rotation of the tibia (Riemann, Myers & Lephart, 2002). Here, the perturbation in a weight bearing condition allows ecological validity of the test (Han et al., 2016) including proprioceptive information from the foot-ankle complex (Han et al., 2015). The fall of the platform was recorded (1,000 Hz) with a triaxial accelerometer (Delsys Inc., Natick, MA, USA) and synchronized with the EMG recording.

### EMG specifications and electrode placement

After shaving and cleaning the skin with alcohol, electrodes were placed on the VM and ST muscles according to SENIAM recommendations (Hermens et al., 1999). For the VM, the recording site was at 80% of the line between the anterior superior iliac spine and the joint space in front of the anterior border of the medial ligament. Electrodes for the ST muscle were located at 50% of the line between the ischial tuberosity and the medial epicondyle of the tibia. Due to the potential for the change in the ST muscle belly following the ACLR, the placement site was carefully corroborated with palpation and a recommended clinical test (Hermens et al., 1999). Single differential amplifiers (DE-2.1, Delsys Inc., Natick, MA, USA) were used to record the EMG signals. In each EMG sensor, there are two silver bar-shaped electrodes with 10 mm × 1 mm contact dimension and 10 mm electrode-to-electrode spacing. EMG signals were pre-amplified in a simple differential manner, recorded at a sampling frequency of 1,000 Hz, and filtered with a bandwidth of 20–450 Hz (Myomonitor IV, Delsys Inc., Natick, MA, USA).

### Testing protocol: perturbation

After verbal consent, subjects were asked to stand barefoot on the platforms, with the lower limb slightly in external rotation (foot progression angle at ∼15°of toe out). The equal distribution of their weight between both extremities was controlled visually at the start of the test to secure a standard starting position. Further uneven weight distribution was not corrected since this could potentially represent the neuromuscular response that the subjects had due to their condition (Soltani et al., 2014). Subjects were instructed to stand on the platforms with the arms hanging loosely at the side and wait for one of the platforms to fall, with the leg that was tested first assigned randomly. The space between the platforms was standardized to keep the feet separated by the width of the shoulders. A perturbation was then generated by the platform with a sudden fall. Seconds later, the system was manually placed in the starting position. After a few demonstrative repetitions, 20 destabilizations were performed randomly alternating the side of destabilization (right/left). Foot position was marked to ensure reproducibility in every attempt and dark glasses and headsets were used to avoid muscle pre-activation and/or anticipation to the destabilization (Fig. 1). During perturbations, the EMG of the analyzed muscles and the acceleration of the platform were recorded.

### Outcome measures

The main outcome of this study was the muscle activation onset time of the VM and ST evaluated with EMG. The average of six onset times (perturbations) was calculated for each muscle of each subject. The onset of EMG activity was determined as follows: signals for data analysis were selected following quality checking proposed by Hodges and Bui for EMG onset detection (Hodges & Bui, 1996). All data with background activity greater than 30% of the mean of the rectified burst in which muscle onset could not be clearly identified were excluded from the analysis. Data in which the EMG onset was masked by movement artefact were eliminated. Signals from at least six destabilizations satisfying these criteria were selected for further analysis.

**Figure 2:**
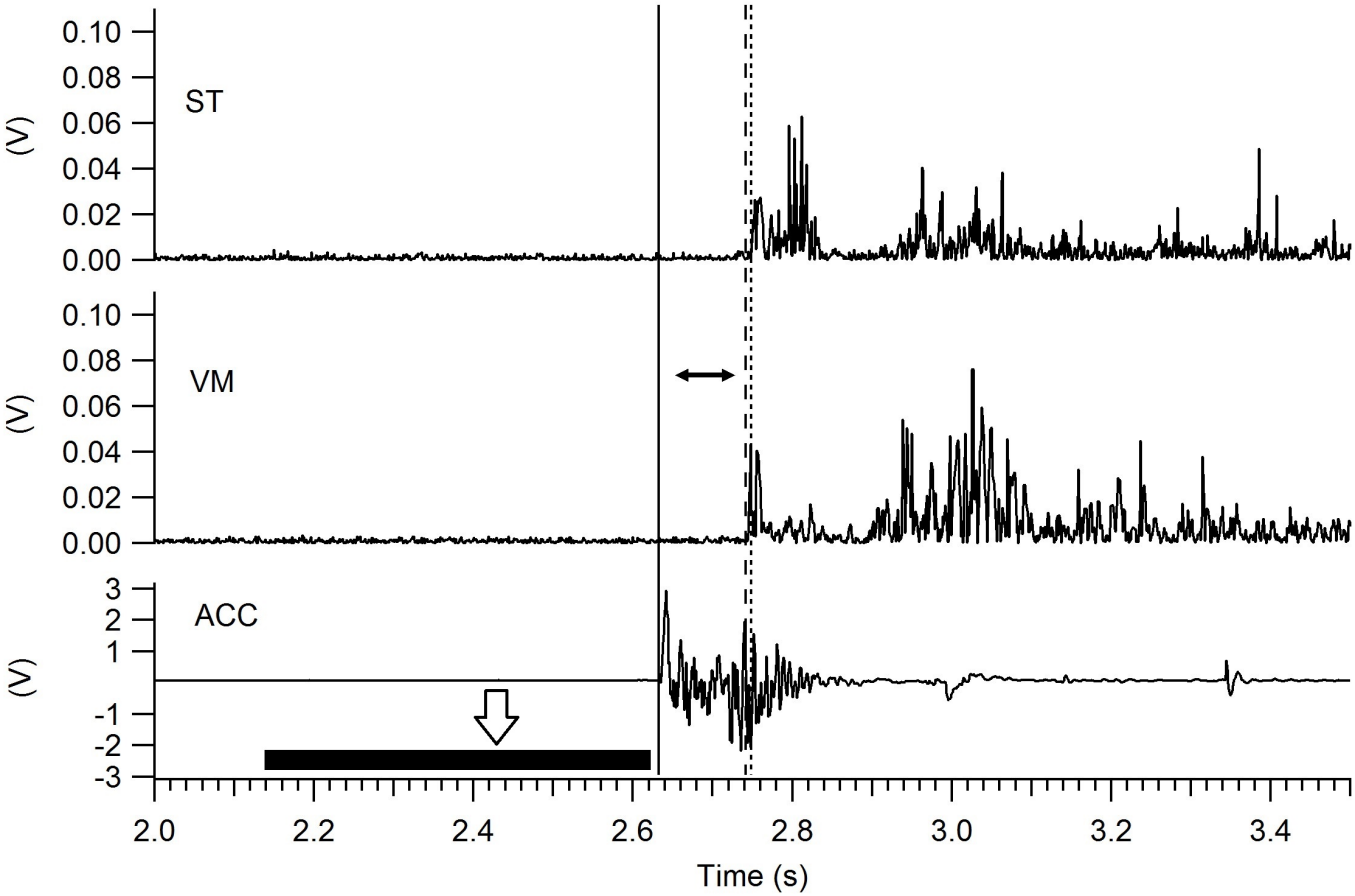
Muscle onset determined by EMG. After the release of the mechanism (white arrow) the first vertical line (left to right) marks the perturbation (impact) of the platform detected with the accelerometer (ACC). The second vertical line (dash) marks when the vastus medialis (VM) activation surpasses the detection threshold, and the third line (dots) represents when the semitendinosus (ST) surpasses the detection threshold. The horizontal black bar represents the 500 ms window for the calculation of the accelerometer threshold (see text for details), similar windows were used for each muscle (not shown in the figure). The muscle onset is defined as the time between the perturbation and the starting of the muscle activation (horizontal black arrow).

Selected EMG traces were full wave rectified and filtered with a digital low-pass filter at 50 Hz. The time between the stimulus (impact of the platform) and the start of muscle activation was calculated and defined as “muscle onset” (Hodges & Bui, 1996). Figure 2 shows the method used to determine the onset for VM and ST. The impact of the platform was detected with the accelerometer and was defined as the time at which acceleration was equal to or higher than the average acceleration of a 500 ms window located before perturbation onset, multiplied by 30 times the standard deviation of the same time window. Muscle activation threshold was determined as the time at which the EMG amplitude was equal to or higher than the average rectified EMG of a 500 ms window located before perturbation onset, multiplied by three times the standard deviation of the same time window (Hodges & Bui, 1996). All signals were processed using custom Igor Pro 6.0 (Wavemetrics, Portland, OR, USA) programs. Both the start of the perturbation and the onset of EMG were determined automatically and visually confirmed by an evaluator (PPM) with experience (4 years). Data processing was performed blinded to group condition.

### Statistical analysis

Because all the ACLR knees were in the preferred limb, the preferred knees in the healthy group were chosen for comparison (same knee side). A two-way mixed ANOVA (repeated measures) with ‘knee side’ (involved/uninvolved) as within-subject factor and ‘group’ (ACLR/Control) as between-subject factor was used to evaluate: (1) differences in muscle onset of the VM and ST muscles between the involved knee of ACLR subjects and uninvolved knee in healthy controls; (2) differences in muscle onset (VM and ST) between the uninvolved knee of the ACLR subjects with the same knee side from uninvolved subjects; (3) differences in muscle onset of the VM and ST between involved and uninvolved knees in the ACLR group. For pair-wise comparisons, *p*-values were adjusted for multiple comparisons according to the Bonferroni adjustment. GraphPad Prism v7 (GraphPad Software, Inc., La Jolla, CA, USA) was used to perform these analyses.

**Table 2:** Pairwise comparisons of muscle onsets of the VM and ST between the involved knee in the ACLR group and the uninvolved knee (same side knee of the control group) *r*.

		Group					95% Confidence interval for difference^a^	
Outcome	Comparison			Mean difference in muscle onset between groups	Std. error	Sig.^a^	Lower bound	Upper bound
VM Onset	Involved knee vs. uninjured knee (same knee side)	ACLR	Healthy	30.800^*^	6.335	<0.001	18.062	43.538
	Uninvolved knee vs. uninjured knee (same knee side)	ACLR	Healthy	26.000^*^	5.594	<0.001	14.753	37.247
ST Onset	Involved knee vs. uninjured knee (same knee side)	ACLR	Healthy	27.120^*^	5.927	<0.001	15.202	39.038
	Uninvolved knee vs. uninjured knee (same knee side)	ACLR	Healthy	31.680^*^	5.853	<0.001	19.912	43.448

**Notes.**

Based on estimated marginal means. ^∗^ The mean difference is significant at the .05 level. ^a^ Adjustment for multiple comparisons: Bonferroni.

ACLR Anterior Cruciate Ligament Reconstruction Group VM Vastus Medialis Muscle ST Semitendinosus Muscle

## Results

**Figure 3:**
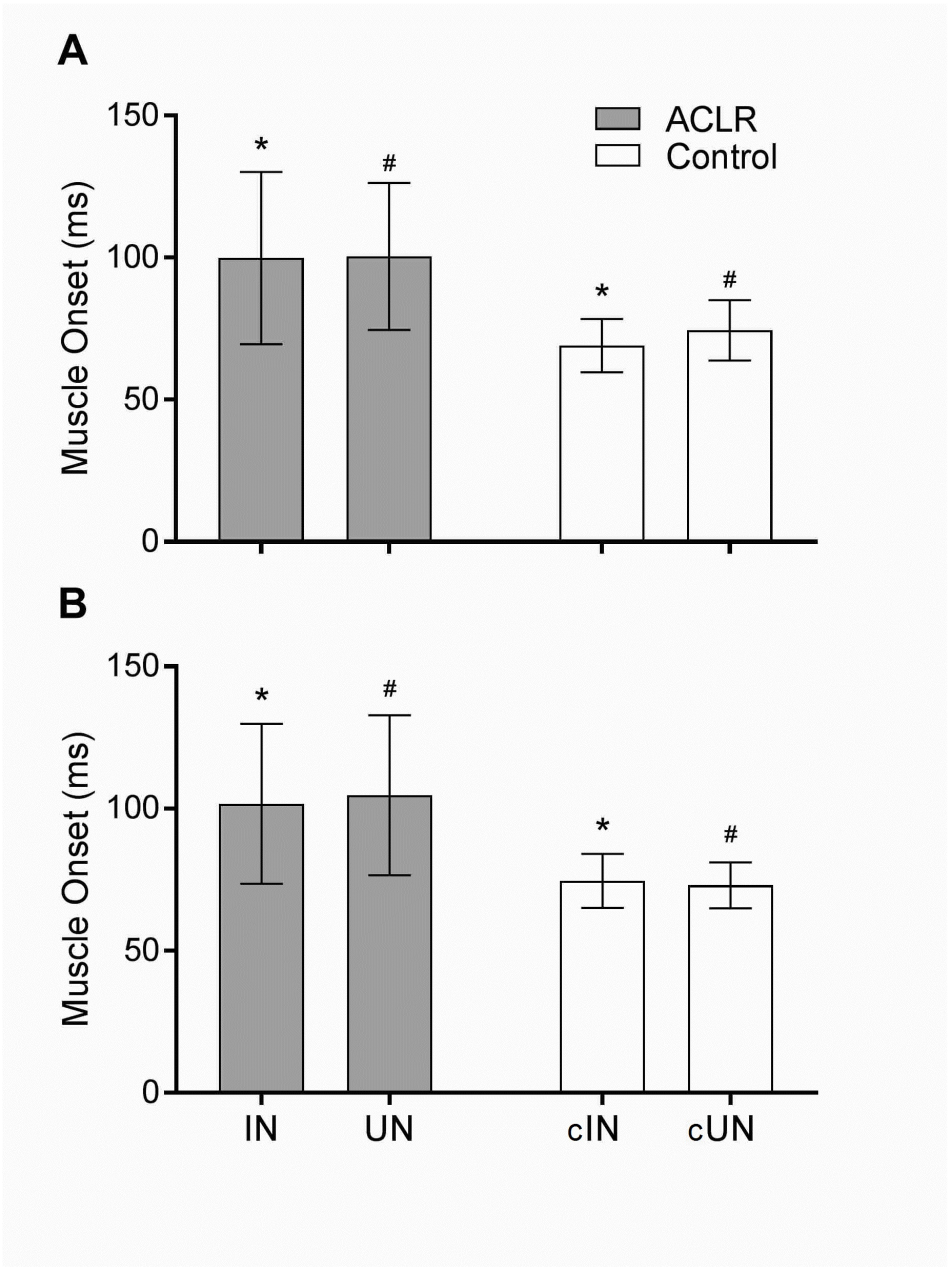
Muscle onset of the vastus medialis (A) and semitendinosus (B) muscles in the anterior cruciate ligament reconstruction (ACLR) and healthy (Control) groups. There was no significant difference between the involved (IN) and uninvolved (UN) knee in the ACLR group. Significant difference was found between the involved (*) and uninvolved (#) knees in the ACLR group with their respective uninjured knee in the control group (cIN and cUN). Error bars represent standard deviations. The level of significance was *p* < 0.05.

The two way ANOVA revealed a significant main effect for ‘group’ in the VM (*F*(1, 48) = 30.7; *P* < 0.01) and ST (*F*(1, 48) = 32.4; *P* < 0.01). However, no main effect was found for ‘knee side’ in either muscle: VM (*F*(1, 48) = 0.93; *P* = 0.34); ST (*F*(1, 48) = 0.06; *P* = 0.8). The Bonferroni contrast (Table 2) found significant differences in EMG onset times of VM (*P* < 0.01) and ST (*P* < 0.01) between the involved knee of ACLR subjects (VM = 99.9 ± 30 ms; ST = 101.7 ± 28 ms) and same knee side in healthy controls (VM = 69.1 ± 9 ms; ST = 74.6 ± 9 ms). Furthermore, significant differences were found for VM (*P* < 0.01) and ST (*P* < 0.01) in the onset times between the uninvolved knee of the ACLR subjects (VM = 100.4 ± 26 ms; ST = 104.7 ± 28 ms) with the same knee side from uninvolved subjects (VM = 74.4 ± 11 ms; ST = 73.0 ± 8 ms). Thus, there was a delayed onset of VM and ST in both knees (involved, uninvolved) in the ACLR group (Fig. 3). No significant differences in the muscle onset for VM and ST were found (*P* > 0.99 and *P* = 0.92 respectively) between the involved (VM = 99.9 ± 30 ms; ST = 101.7 ± 28 ms) and uninvolved knee in the ACLR group (VM = 100.4 ± 26 ms; ST = 104.7 ± 28 ms), indicating a bilateral alteration of the muscle onset (Table 3, Fig. 3).

**Table 3:** Pairwise comparisons of muscle onsets of the VM and ST between the involved and uninvolved knee in the ACLR group.

						95% Confidence interval for difference	
Outcome	Comparison	Group	Mean difference in muscle onset between legs	Std. Error	Sig.	Lower bound	Upper bound
VM Onset ST Onset	Involved knee vs. Uninvolved knee	ACLR	−0.560	4.348	0.898	−9.303	8.183
	Involved knee vs. Uninvolved knee		−3.000	4.008	0.458	−11.058	5.058

**Notes.**

ACLR Anterior Cruciate Ligament Reconstruction Group VM Vastus Medialis Muscle ST Semitendinosus Muscle Involved knee: knee that underwent surgery

## Discusion

Our results show that subjects with ACLR not only present a delayed onset of the studied muscles in the involved knee, but also in the uninvolved healthy knee. This impairment is evident after a stimulus applied at the ankle joint.

Our first finding was that subjects after ACLR surgery had a significantly delayed muscle onset of the VM and ST when compared with healthy subjects. These results are consistent with studies investigating subjects with ACL injury (Madhavan & Shields, 2011), which revealed longer latency and altered EMG profile of the VM specifically when compared with control groups. Further this finding is in accordance with a previous study that examined muscle activation onset times during the transition from double-leg stance to single-leg stance in ACLR subjects (Dingenen et al., 2016). A possible explanation for these delays could be an altered sensitivity of the muscle spindles due to changes in afferent input. As suggested by Riemann & Lephart (2002) afferent signals coming from muscles, ligaments, skin, joint tissues, and descending commands from supraspinal areas converge and have an influence on the activity of dynamic and static gamma motor neurons. Since ACLR and the subsequent rehabilitation process lead to a change in the proprioceptive information around the knee (Johansson, Sjölander & Sojka, 1991), this altered input could affect the function of the gamma system. Because the activity of the gamma system alters muscle spindle sensitivity and pre-activation, it regulates in part the activation of extrafusal fibers, perhaps leading to an altered muscle onset. Further, a review of the biomechanical changes that occur after an ACLR showed that although reconstruction restores antero-posterior stability of the knee, it does not completely restore rotational stability (Pappas et al., 2013). This could change the regular arthrokinematics of the knee, further altering the afferent signal from mechanoreceptors around the joint. Thus, the altered muscle onset timing present in the involved knee may represent a modification of neuromuscular control facilitated by an altered sensitivity of muscle spindle gamma motor neurons.

Our second finding was a delay in muscle onset of the uninvolved knee in the ACLR group. These results are similar to those of previous studies (Lustosa et al., 2011; Dingenen et al., 2015; Dingenen et al., 2016), who described that subjects with ACL injury and reconstruction presented bilateral alteration in muscle activation during different functional tasks. Two possible theories may explain these findings and our results. First, bilateral onset alterations can be explained by the aforementioned abnormal gamma loop activity after the reconstruction, in this case affecting the uninvolved side of the ACLR patients. The altered proprioceptive input in the reconstructed knee may evoke contralateral fusimotor reflexes which are potent enough to significantly change the sensitivity of primary and secondary muscle spindle afferents in the uninvolved knee (Djupsjöbacka et al., 1995). It is argued that secondary spindle afferents project back to *γ* motoneurones innervating spindles in ipsi- and contralateral muscles (Sjölander, Johansson & Djupsjöbacka, 2002). This intrinsic network mediates sensory feedback which is particularly important for bilateral muscle coordination (Sjölander, Johansson & Djupsjöbacka, 2002). Further, our results suggest that this response can be elicited in different parts of the proprioceptive network (e.g., the ankle), not only in the affected zone.

The second possible explanation for the bilateral neuromuscular impairment is related with changes in the CNS following injury and reconstruction. Early studies observed bilateral quadriceps inhibition in cases of unilateral ACL injury, suggesting a crossover mechanism, probably caused by decreased descending activation within the CNS (Urbach et al., 1999; Urbach & Awiszus, 2002). Recent studies have shown that ACL injury, reconstruction and rehabilitation may cause increased activation of motor, visual, and secondary sensory areas in the brain, compared to uninjured subjects (Grooms et al., 2017). These brain activation differences indicate a possible neuroplastic effect of musculoskeletal trauma that is not normalized after treatment or return to activity (Grooms, Appelbaum & Onate, 2015; Grooms et al., 2017; Needle, Lepley & Grooms, 2017). Indeed, ankle proprioception-specific neural activity links with brain activity during balance performance while standing (Goble et al., 2011). Hence, CNS reorganization may be a suitable explanation for the persisting neuromuscular impairments presented here after ankle perturbations (Ward et al., 2015; Dingenen et al., 2015; Dingenen et al., 2016).

Dingenen and colleagues, demonstrated a bilateral impairment of muscle onset times in patients with ACLR reconstruction during the transition from double leg stance to single-leg stance (Dingenen et al., 2016). This delayed neuromuscular response when transitioning may be due to a neurocognitive overload secondary to CNS reorganization (Dingenen et al., 2016). While the authors argue that this kind of adaptation is better to explain altered muscle onsets rather than altered afferent information coming from the injured joint (Dingenen et al., 2016), the nature of their task is different from our unexpected perturbations. It has been suggested that in comparison to multi-planar tasks, single planar-unexpected perturbations produce greater horizontal center of mass displacements and peak accelerations, which may elicit greater neuromuscular responses (Malfait et al., 2015), In this kind of perturbation, movement control relies more on proprioceptive information (Han et al., 2016), in comparison with planned tasks where attentional demands have a detrimental impact on ankle joint proprioceptive performance (Yasuda et al., 2014). On the whole, it may be the case that both hypotheses presented here—abnormal gamma loop activity and CNS neuroplastic changes—are suitable to explain the bilateral neuromuscular alterations seen in this study.

It is possible that the altered muscle activation patterns were already present before the injury. It has been suggested that neuromuscular deficits may predispose knee injury (Read et al., 2016). Further, a previous case study suggests that delayed muscle onset may be a risk factor for ACL injury (Saunders et al., 2014). This remains unclear based on the retrospective design of our study. While a recent systematic review and meta-analysis (Theisen et al., 2016) suggests that muscle activity onset is not associated with increased ACL injury risk, current evidence is still scarce and weak (Theisen et al., 2016). Future prospective studies should examine whether neuromuscular deficits and altered muscle onsets are present before ACL injuries.

Although most of the evidence in the previous literature agrees with our results, Kalund and colleagues (Kalund et al., 1990) showed quicker onset responses of subjects with ACL deficiency when compared with a healthy group. Further, Beard and colleagues demonstrated significant differences in the onset timing of hamstring muscles between involved and uninvolved limb (Beard et al., 1994). These discrepancies may be explained by the different methodologies used. In their research, Kalund and colleagues investigated the EMG onset timing of hamstrings and quadriceps during uphill walking. They demonstrated that with a mechanical loss, the system may initiate a strategy to generate onsets earlier to adapt to this mechanical deficit. However, this kind of feed-forward onset strategy cannot be compared to our testing. Moreover, their study included subjects with unreconstructed ACL injuries and longer periods from diagnosis to evaluation, which make direct comparisons difficult. Beard and colleagues demonstrated differences in the reflex latency of the hamstring muscles between ACL involved and uninvolved limbs. However, they used a model of postero-anterior shear force applied directly over the knee, while also examining ACL deficient subjects. A weight bearing situation with unexpected perturbations helps to reveal proprioceptive deficiencies at the lower limb, as the lower limb acts as a linked segment model, where distal single plane translational perturbations elicit large postural responses (Malfait et al., 2015). Thus, the methodology presented here provides a way of generating unpredictable changes to the environment that can be linked to neuromuscular activity patterns during real sports situations (Oliveira et al., 2012; Malfait et al., 2015)

In our sample, we evaluated the ST muscle, which was directly affected by the surgery. It has been demonstrated that muscle and tendon properties are affected after harvesting the muscle for the graft (Tadokoro et al., 2004). Thus, we acknowledge the possibility that the onset pattern of the ST in our sample was directly affected by muscle damage produced by the surgical preparation. However, based on our results, we believe that the direct impact that the harvest site has on onset patterns is at least equivocal. Vairo and colleagues (Vairo et al., 2007) showed that semitendinosus-gracilis grafts did not significantly alter neuromuscular performance of the semitendinosus muscle (including reactive muscle activation) nor biomechanical strength/endurance qualities during a landing task. It is important to note that we did not find differences between the involved and uninvolved knees for ST muscle onset. This may indicate that ST muscle onset is not necessarily affected by the graft 9 months after surgery, but it is affected by the neuromuscular state of the knee. Thus, we believe that the graft site cannot be considered as the main cause of the EMG onset alteration in the ST found in this study.

### Clinical Implications

Previous research (Crow, Pizzari & Buttifant, 2011) has suggested that dysfunction of muscle onset times may persist in asymptomatic subjects even when some clinical indicators are considered normal. In addition, persistence of these altered onset patterns may contribute to recurrence of injury. Thus, it is important to address these muscle impairments early in the rehabilitation process to improve muscle performance.

Our results indirectly suggest that CNS reorganization and altered modulation of afferent information should be the target of rehabilitation. While specific interventions may address each of this components (e.g., transcutaneous nerve stimulation for spinal-reflex–inhibitory pathways (Pietrosimone et al., 2009), and transcranial magnetic stimulation for corticomotor excitability (Gibbons et al., 2010), we do not know how to most effectively intervene in an interconnected neural system to improve motor output (Pietrosimone et al., 2015). Moreover, individually targeting either the afferent-spinal reflex–or corticomotor-excitability pathways with a treatment may have an indirect effect on the other (Pietrosimone et al., 2015). Thus, therapeutic interventions should be broader and target CNS re-education, and may focus on coordinated multi-segmental tasks when loading the lower limbs to mimic real sport situations (Dingenen et al., 2016). Progressively challenging functional exercises in conditions of uncertainty, with an external focus of attention (Benjaminse et al., 2015), and involving the entire lower extremity and both legs, may help to facilitate the development of automatic unconscious anticipatory muscle activation patterns (Dingenen et al., 2015; Dingenen et al., 2016). However, further studies are needed to elucidate the most effective exercise prescriptions to alter impaired muscle activation patterns.

### Limitations

All participants were amateur soccer players, so these results may not be generalizable to other populations. Although there were no statistical differences in demographics between groups, it could still be possible that other factors not investigated in this study (e.g., muscle strength or inhibition, rehabilitation protocol, ankle biomechanics) could have influenced the EMG onset of subjects with ACLR.

Despite the fact that the functional status of our ACLR group was assessed with functional and clinical tests, a more objective measurement (e.g., a validated questionnaire, patient-reported outcome measures) may be desirable to assess knee function. The perturbation method implemented may not be considered specific for the ACL compared to other proposed methods in the literature (Beard et al., 1994; Madhavan & Shields, 2011). However, distal perturbations in a weight bearing position can evoke clear neuromuscular responses on the whole lower limb (Malfait et al., 2015; Kazemi et al., 2017). This method intended to provide a stimulus to the lower limb in a weight bearing position, ensuring activation of muscles, joint capsule compression and skin stretch during the evaluation (Shultz et al., 2000; Han et al., 2016), information that is crucial for central proprioception and balance control (Riemann & Lephart, 2002; Han et al., 2015; Han et al., 2016). Only two EMG signals per leg were evaluated in this study. Supplementary EMG signals from more muscles on the lower limb are desirable to discuss whether the finding of bilateral onset alteration is present in all lower limb muscles or just those around the knee. While other studies have demonstrated bilateral altered muscle onset times on the ankle, knee and hip of ACLR subjects during a transition task (Dingenen et al., 2016) further studies are needed to elucidate whether this alteration is present after single-planar unexpected perturbations around the ankle.

## Conclusions

Amateur soccer players with ACLR present delayed VM and ST muscle onset in the involved and uninvolved knees in comparison to uninjured knees of healthy subjects. This may suggest a bilateral neuromuscular impairment dependent on central processing of proprioceptive information and/or CNS reorganization.

##  Supplemental Information

10.7717/peerj.5310/supp-1 Data S1 Raw data

## Additional Information and Declarations

### Data Availability

The following information was supplied regarding data availability: Raw data and supplemental information of patients in the study group are provided in Data S1.
